# Molecular characterization of strains of *Zymomonas mobilis *by sequencing the 16S ribosomal

**DOI:** 10.1186/1753-6561-8-S4-P236

**Published:** 2014-10-01

**Authors:** Livia Caroline Araujo, Tayane Mendes, Márcia Silva

**Affiliations:** 1 Dept. de Bioquímica, Universidade Federal de Pernambuco, Recife, PE, Brazil

## Background

*Zymomonasmobilis *has attracted great interest in the scientific, industrial and biotechnological due to its high potential fermentation. From the viewpoint of taxonomic *Z. mobilis *is the only species of the genus *Zymomonas *, and is subdivided into three subspecies: *Z. mobilis *subsp. *mobilis, Z. mobilis *subsp. *pomaceae *and *Z. mobilis *susp. *francensis*. Differentiation between these three subspecies is based on physiological tests. These tests are time consuming and often unreliable. Therefore, molecular techniques are proposed as a quick and reliable way to characterize the genetic variability of these bacteria. This study aimed to perform molecular characterization of 6 strains of *Zymomonasmobilis *deposited in the Collection of Microorganisms of Department of Antibiotics, Federal University of Pernambuco (UFPEDA).

## Methods

The strains were grown in SSDL for 24 hours at 30 °, followed by centrifugation and extraction of chromosomal DNA. PCR reactions were performed using specific primers and conditions for amplification of the *16S rDNA*. The products of the amplified *16S rDNA *were purified and sequenced using a ABI PRISM^® ^3500 Genetic Analyzer (Applied Biosystems) at the Sequencing Platform LABCEN/CCB in the Universidade Federal de Pernambuco (Recife, Brazil). The data obtained by sequencing the *16S rDNA *were analyzed and compared by BLASTn programs and aligned by MultiAlin.

## Results and conclusion

The sequences obtained were different degrees of similarity to strains from international collections. Multiple alignment of the sequences of the *16S rDNA *of strains of *Z. mobilis *UFPEDA gene regions revealed a high degree of conservation. Sequence analysis of the *16S rDNA *confirmed that all the strains belong to the species *Zymomonas mobilis *. However, it was not possible to differentiate the level of subspecies. From the results obtained by multiple alignment of the sequences was possible to prove the stability and degree of conservation of all lineages. Based on these results, other phylogenetic markers should be used to better characterize the genetic variability of strains of *Z. mobilis *deposited at the collection UFPEDA.

